# Transcriptional Effects of Ozone and Impact on Airway Inflammation

**DOI:** 10.3389/fimmu.2019.01610

**Published:** 2019-07-10

**Authors:** Sharon Mumby, Kian Fan Chung, Ian M. Adcock

**Affiliations:** Respiratory Section, National Heart and Lung Institute, Imperial College London, London, United Kingdom

**Keywords:** gene expression, immune cell recruitment, acute ozone exposure, chronic ozone exposure, pro-inflammatory signaling

## Abstract

Epidemiological and challenge studies in healthy subjects and in individuals with asthma highlight the health impact of environmental ozone even at levels considered safe. Acute ozone exposure in man results in sputum neutrophilia in 30% of subjects particularly young children, females, and those with ongoing cardiopulmonary disease. This may be associated with systemic inflammation although not in all cases. Chronic exposure amplifies these effects and can result in the formation of asthma-like symptoms and immunopathology. Asthmatic patients who respond to ozone (responders) induce a greater number of genes in bronchoalveolar (BAL) macrophages than healthy responders with up-regulation of inflammatory and immune pathways under the control of cytokines and chemokines and the enhanced expression of remodeling and repair programmes including those associated with protease imbalances and cell-cell adhesion. These pathways are under the control of several key transcription regulatory factors including nuclear factor (NF)-κB, anti-oxidant factors such as nuclear factor (erythroid-derived 2)-like 2 NRF2, the p38 mitogen activated protein kinase (MAPK), and priming of the immune system by up-regulating toll-like receptor (TLR) expression. Murine and cellular models of acute and chronic ozone exposure recapitulate the inflammatory effects seen in humans and enable the elucidation of key transcriptional pathways. These studies emphasize the importance of distinct transcriptional networks in driving the detrimental effects of ozone. Studies indicate the critical role of mediators including IL-1, IL-17, and IL-33 in driving ozone effects on airway inflammation, remodeling and hyperresponsiveness. Transcription analysis and proof of mechanisms studies will enable the development of drugs to ameliorate the effects of ozone exposure in susceptible individuals.

## Introduction

It has been known since the mid-1980s that ozone exposure induces airway inflammation and reduces lung function (1). As highlighted elsewhere, ozone is a powerful gaseous oxidant and toxic air pollutant that is inhaled and thereby affects the lung (2). The highly reactive oxidative agent ozone, or catena-trioxygen (O_3_), is consumed by protective processes within the epithelial lining fluid to produce secondary oxidation products that activate the airway epithelium to enhance inflammatory signaling pathways and induce several pro-inflammatory and immune factors (2). In this Review, we will discuss the effects of ozone on the activation of pro-inflammatory pathways in airway epithelial cells. We then discuss the epidemiological effects of ozone and that of controlled ozone-exposure on clinical and immune features in healthy subjects and in at-risk subjects including asthmatics and patients with other airways diseases and those in the youngest and oldest populations. Finally, we discuss how acute and chronic ozone-exposure models may provide insight into the clinical effects of ozone. The data also highlights the importance of acute vs. chronic exposure and also the effect of the timing of samples collection following ozone exposure for the analysis of ozone effects.

## Mechanisms Implicated in Ozone Actions on the Airway Epithelium

There are differences in the responses of cells and animals to particulate matter (PM) and ozone (3). Both PMs and ozone can activate membrane receptors, intracellular kinases particularly NF-κB and phosphatases, and transcription factors that regulate inflammatory responses. Understanding these mechanistic processes will allow both preventative sand therapeutic strategies to be designed for subjects exposed to ozone for prolonged periods.

**Figure 1:**
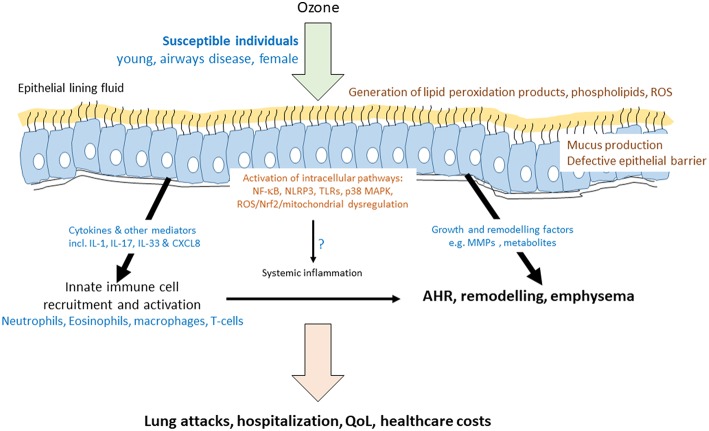
Mechanisms of ozone-induced transcriptome activation in the airway. Schematic diagram indicating that ozone dissolves in the epithelial lining fluid of susceptible individuals to produce reactive oxygen species (ROS) and modified lipids. These act on the epithelial cells to activate a number of key intracellular and cell surface pathways leading to the induction of the mRNA for cytokines, growth factors, and remodeling enzymes. As a result, there is an acute effect on the recruitment and activation of innate immune cells and on epithelial barrier function and mucus production. These acute effects of ozone may impact on the levels of inflammatory mediators and cells in the blood. Airway inflammation may be directly, or indirectly, associated with airway hyperresponsiveness (AHR). Prolonged ozone exposure has a greater remodeling effect and can result in emphysema. Together, both acute and chronic ozone exposure results in increased hospitalisations due to lung exacerbations or attacks, decreased quality of life (QoL) in at-risk individuals and a large healthcare cost for the individual and for society.

Mechanistically, inhaled ozone does not enter cells but reacts with components of the airway lining fluid to generate other reactive oxygen species (ROS) to enhance local oxidative stress, inflammation, and epithelial cell injury (4) (Figure 1). Recent data also highlights a pro-inflammatory role for ozone-derived oxysterols (epoxycholesterol-α and -β and secosterol A and B) (5). These form in the airway lining fluid and form lipid-protein adducts with the liver X receptor (LXR) resulting in suppression of cholesterol synthesis pathways. Ozone-induced lung injury pulmonary inflammation has been linked to altered expression of pro-resolving lipid mediators (SPMs) such as 14-HDHA and 17-HDHA and the SPM protectin DX (PDX) in the lung of male C57Bl/6J mice (6). Exogenous administration of 14-HDHA and 17-HDHA prior to ozone exposure decreased proinflammatory cytokine and chemokine expression, and decreased BAL macrophages and PMNs. Ozone also enhances the expression of several eicosanoids and oxidized lipids including 20-hydroxyeicosatetraenoic acid (20-HETE) in the BAL of ozone-exposed animals. These oxidized lipids may mediate the effects of ozone on AHR *in vivo* as demonstrated by effects on precision cut lung slices (7). In contrast, prostaglandin E2 (PGE2) may not be important since knockout of microsomal prostaglandin E synthase-1 (mPGES-1) did not impact on ozone-induced AHR or inflammation (8).

###  Key Role of Oxidative Stress in Ozone Actions

The critical role of oxidative stress in ozone function was highlighted by the fact that lipid oxidation, epithelial proliferation, bronchial mucous cell hyperplasia, and mucus hypersecretion were greater in Nrf2-/- than in wild type mice (9). Furthermore, the potent and selective Nrf2 activator 3-(pyridin-3-ylsulfonyl)-5-(trifluoromethyl)-2H-chromen-2-one (PSTC) restored ozone-induced glutathione depletion with no off-target inhibition of IL-1β-induced NF-κB translocation in human bronchial epithelial cells (HBECs) (10). Interestingly, the serum innate immune protein, mannose-binding lectin (MBL), helps drive ozone-induced proinflammatory events. Ozone (0.3 ppm)-exposed MBL-deficient (Mbl-/-) mice produced significantly reduced levels of BAL inflammatory markers including eosinophils, neutrophils, CXCL2 and CXCL5, and gene array analysis revealed differences in the NRF2 signaling networks (11).

In addition, high doses of the anti-oxidant N-acetylcysteine (NAC) prevents and reverses ozone-induced lung damage *in vivo* (12). NAC given prophylactically prevented ozone-induced BAL inflammation (macrophages) and airway smooth muscle (ASM) mass whilst NAC given therapeutically reversed AHR in addition to reducing ASM mass and the number of apoptotic cells.

### Differences Between *in vivo* and *in vitro* Effects of Ozone on Cellular and Mitochondrial ROS

There are controversies regarding the role of ROS in mediating ozone functions both *in vitro* and *in vitro*. It is possible that mitochondrial damage leading to oxidant stress may play an important role in the pathogenesis of airflow obstruction and emphysema following ozone exposure (13). Mice exposed to ozone have mitochondrial dysfunction reflected by decreased mitochondrial membrane potential (ΔΨm), increased mitochondrial oxidative stress, and reduced mitochondrial complex I, III, and V expression. This was associated with airway inflammation and AHR (14). Pharmacological reversal of mitochondrial dysfunction by the mitochondria-targeted antioxidant MitoQ reduced inflammation and AHR. In another study, a similar mitochondrial-directed anti-oxidant, MitoTEMPO, reduced chronic (6 week) ozone-induced (2.5 ppm, 3 h, twice weekly) lung inflammation (BAL cells and IL-1β, KC/CXCL1 and IL-6) and oxidative stress but had no effect on emphysema (Lm) scores. In contrast, inhibition of the NLPR3 inflammasome, which is activated by mitochondrial ROS (mtROS) and other stimuli, suppressed ozone-induced inflammation, oxidative stress, emphysema, airway remodeling, and airflow limitation (Figure 1).

Further evidence for a role of mitochondrial dysregulation in ozone-induced inflammation and AHR was shown by restoration of defective mitochondria using induced pluripotent stem cell-derived mesenchymal stem cells (iPSC-MSCs) *in vitro* and *in vivo* (15). iPSC-MSCs protected against oxidative stress-induced mitochondrial dysfunction in human ASMCs and in mouse lungs while reducing airway inflammation and AHR.

### Toll-Like Receptors (TLRs) and Other Cell Surface Receptors Mediating Ozone-Induced Inflammation

Ozone also impacts on AHR and neutrophilic inflammation though activation of Toll-like receptor (TLR) 2 and TLR4 (16). In addition, there is a priming effect of ozone on the innate immune response as exemplified by enhanced TLR2 and TLR4 expression and functional responses to their respective agonists (17, 18). Ozone alone enhanced TLR4, TLR2, and TLR1 expression on macrophages which may account for the ability of subsequent challenge of ozone-exposed mice to Pam3CYS, a synthetic TLR2/TLR1 agonist, to enhance lung IL-6 and KC/CXCL1 and reduce TNFα and MIP1α expression. Transcriptomic and bioinformatic analysis of lung tissue revealed significant effects on innate immune pathways. Of interest, hyaluronan, a damage-associated molecular pattern essential for the full range of ozone effects seen *in vivo*, acts through the TLR4/MyD88-TIRAP pathway (19, 20). The primary receptor for hyaluronic acid (HA), which is important in lung injury and is elevated following ozone exposure, is CD44. Genetic experiments using CD44 deficient mice or those lacking inter-alpha-trypsin inhibitor, which facilitates HA binding, do not demonstrate AHR following ozone exposure. In addition, pharmacologic pretreatment of ozone-exposed mice with HA-binding peptide protects against ozone-induced AHR whilst HA itself enhances the effect of ozone in a CD44-dependent manner (21). Together, these results suggest that the extracellular matrix is important in ozone-induced AHR.

Genetic deletion of surfactant protein-D (SFTPD), a collectin important for suppressing macrophage inflammatory responses alters the response to ozone (22). In SFTPD(-/-) mice, there was increased presence of lung injury and oxidative stress and the increased numbers of BAL macrophages were enlarged and foamy. SFTPD(-/-) mice also presented with a worsening of central airway and parenchymal mechanics consistent with the loss of parenchymal integrity. Ozone modulation of SFTPD regulates an IFNγ/IL-12 feedback loop which amplifies dendritic cell homing to mediastinal lymph nodes (23).

### Intracellular Signaling Pathways Mediating Ozone Actions

There is evidence that p38 mitogen-activated protein kinase (MAPK) pathway mediates ozone-enhanced airway inflammation and remodeling as well as AHR in murine models of asthma (24). These responses to ozone are relatively corticosteroid insensitive but combination of dexamethasone with a p38 MAPK inhibitor (SB239063) suppressed ozone effects on inflammation and AHR suggesting that p38 MAPK activation is involved in the corticosteroid insensitivity seen in this model. SB239063 alone prevented ozone-induced airway resistance (Raw), lung compliance (CL), and BAL IL-13 levels when given in the context of an ovalbumin challenge (25). In addition, the anti-oxidant α-tocopherol alone reduced BAL eosinophils and macrophages and peribronchial inflammation. Both drugs also prevented increases in ozone-induced AHR, BAL IFNγ, and IL-6 expression and perivascular lung inflammation. This effect was enhanced using a combination of both drugs indicating a role for both p38 MAPK and oxidative stress in ozone-induced exacerbations of asthma.

The p38 MAPK pathway is also involved in the mechanism of action of the novel gaseous transmitter hydrogen sulfide (H_2_S), which partially reversed ozone-induced lung inflammation, oxidative stress and emphysema (26, 27). Other pathways targeted by H_2_S include the Akt pathway and the NLRP3-caspase-1 system. Other MAPK pathways may also be important since the effect of OBC on lung inflammation was dependent on the activation of MAP4K4 in CD4(+) T cells (28).

A number of other signaling pathways have been implicated in ozone-induced inflammation, tissue damage and AHR. The spleen tyrosine kinase (Syk) inhibitor NVP-QAB-205 significantly reduced AHR in an ovalbumin-exposed and challenged mouse model of asthma and restored the enhanced response to PM2.5/ozone back to normal levels (29). Furthermore, IL-10 overexpression protects mice from the detrimental inflammatory, NF-κB-mediated effects of ozone (30). The EGFR kinase inhibitor PD153035, given prophylactically, prevents ozone-induced EGFR (Y1068) phosphorylation in the lung sections and significantly attenuates lung inflammation (31). The effects of therapeutic administration on inflammation and lung function should be measured.

The airway remodeling induced by ozone exposure may involve the presence of neutrophil-derived neutrophil gelatinase-associated lipocalin (NGAL) (32). The effects seen *in vivo* on the suppression of E-cadherin and up-regulated α-SMA expression were replicated by NGAL exposure in 16HBE cells acting through the WNT/glycogen synthase kinase-3β (GSK-3β) pathway.

Ozone also impacts on cardiorespiratory conditions including hypoxia-associated pulmonary hypertension (HPH) which often occurs in COPD (33). Male C57BL/6 mice exposed to 3 weeks hypoxia (10.0% O_2_) followed by ozone (4 h, 1 ppm) resulted in increased inflammation, oedema, and AHR. Fasudil, a Rho kinase inhibitor, reduced pulmonary endothelial barrier damage suggesting that enhanced pulmonary vascular pressure may contribute to lung injury, inflammation, and oedema following ozone exposure (34). In addition, the hypoxia induced transcription factor HIF-1α mediates MIF effects on ozone-induced BAL cell counts, cytokine, and AHR (35).

## Epidemiological Impact of Ozone in Healthy and at Risk Subjects

Controlled human exposure to ozone causes decrements in lung function, increased lung neutrophilia and increased airway levels of pro-inflammatory cytokines (4). It is not surprising, therefore, that air pollution is a known asthma trigger and has been associated with short-term asthma symptoms, airway inflammation, decreased lung function, and reduced response to asthma rescue medications and increases the risk of asthma hospitalizations and healthcare utilization (36). In addition, traffic-related pollutants may be causally related to childhood asthma (36).

Ozone impacts equally upon both FEV_1_ and FVC and therefore does not result in a decrease in FEV_1_/FVC. This effect is age-dependent with an increased risk in susceptible populations particularly the very young, pregnant women and individuals with an existing cardiopulmonary disease (37). Acute airway injury and inflammation also results from ozone exposure including enhanced localization of neutrophils and increased cytokine and chemokine expression (1). An early observational study in 38 asthmatics and 13 healthy control subjects in metropolitan Atlanta showed that ozone induced more symptoms of upper airway disease in asthmatics (38). High ozone levels were associated with greater airflow obstruction, lower asthma quality of life scores, more eosinophilia, and higher exhaled nitric oxide (NO) levels in asthmatics. These associations were enhanced in atopic participants irrespective of asthma status. Peak Expiratory Flow (PEF) levels were also negatively associated with weekly average ozone levels for 605 children in Turkey without upper respiratory tract complaints (39).

Significant increases in sputum neutrophilia is only seen in ~30% asthmatics (40). This mimics other markers of sputum neutrophil activation and exhaled breath malondialdehyde (MDA) levels. There was a significant correlation between the ozone-induced fall in FEV_1_ and sputum neutrophil numbers. In addition, there was evidence for sputum eosinophilia post ozone exposure (40). Furthermore, ozone significantly increased neutrophil numbers and myeloperoxidase levels in bronchoalveolar lavage and produced a 4-fold increase in bronchial mucosal mast cell numbers (41). Asthmatic patients not previously treated with inhaled corticosteroids (ICS) and those with a lower FEV1 were more likely to respond to ozone (Figure 1). Lower baseline airway inflammation, younger age and greater airway hyperresponsiveness (AHR) was associated with airway neutrophilia in response to ozone (42). It is possible, therefore, that the lung function and inflammatory responses to ozone are different.

### Ozone Increases the Risk of Emergency Room Visits

The risk of increased emergency room visits in subjects with acute respiratory infections, asthma, chronic obstructive pulmonary disease (COPD), and pneumonia in response to environmental ozone exposure across 17 US states was also age related (43). There is evidence that ozone exposure causes systemic inflammation as measured by blood club cell protein (CCP)-16 levels (44). Recent studies examining data from 6,488 subjects from the National Health and Nutrition Examination Survey (NHANES) between 2005 and 2006 demonstrated a positive association between ozone exposure and emergency room visits in asthmatic patients (adjusted OR 1.07, 95% CI: 1.02–1.13). However, this effect was less than that seen with PM_2.5_ and NO_2_ exposure (45). In addition, time-series analyses of ozone exposure and emergency room visits across 17 States in the USA demonstrated a significant increase in the rate of admission per 20 ppb increase in ozone across all age groups. The effect was greater in adults compared to children and in subjects with asthma and COPD (43). Finally, short term exposure of asthmatics to increasing levels of ozone in the Chinese province of Hubei resulted in asthma mortality (46).

It is evident that ozone exposure also impacts upon the health status of patients with other respiratory diseases such as idiopathic pulmonary fibrosis (IPF) and adult respiratory distress syndrome (ARDS). For example, chronic exposure to ozone enhances the risk of ARDS among older adults (>65 years of age) in the USA (47). Increases of 1 ppb in annual average ozone levels was associated with increases in annual hospital admission rates for ARDS of 0.15% (95% CI, 0.08–0.22) which was evident even in low-pollution regions (annual average ozone level <45 ppb) below the current annual US National Ambient Air Quality Standards. Furthermore, long term exposure (3 years) to ozone was significantly (*P* < 0.01) associated with the development of ARDS following acute trauma even at exposure levels generally below European and USA air quality standards (48).

In a French IPF cohort (COhorte FIbrose, COFI), increasing ozone levels measured close to the patient's home was associated with acute exacerbations (AE) which are associated with high mortality (49). However, in this study ozone did not affect mortality although this was increased by increased exposure to PM2.5 and PM10. In an earlier study, AEs were significantly associated with increases in ozone exposure in the 6 weeks prior to hospital admission (50). Although air pollution *per se* is associated with AEs, disease progression and mortality in IPF, ground level ozone exposure over 40 weeks does not affect lung function (51). Overall, the data suggest that elevated levels of ozone increase respiratory tract complaints with a greater effect seen in susceptible adults and in children.

Ozone also impacts on the health of non-respiratory patients and the risk of mortality is increased in sepsis patients exposed to environmental ozone (52). The mechanisms for this effect was unclear, however, in 3,820 non-current smoking subjects from the Framingham Heart study, there was a negative correlation between ozone exposure and systemic monocyte chemoattractant protein (MCP)-1 levels as a marker of systemic inflammation (53). It is of interest that many of these exposures that affect AEs, for example, are below current National Guidelines which has huge public health implications.

### Controlled Exposure to Ozone: Effects on Lung Function and Immune Cell Activation

Early studies examining the acute effect effects of O3 (220 ppb, 2.25 h) exposure under laboratory conditions on respiratory physiology in healthy subjects revealed an acute effect on lung function parameters such as FEV1 and FVC and effects on AHR to methacholine (Mch) and on respiratory epithelial permeability at 1-day postexposure (54). The responder profiles to these different readouts differed in that either the acute decreases in FEV1 or the development of AHR or epithelial permeability at 1 day post ozone did not always coincide suggesting that these are separate and independent phenotypic effects seen with O3 exposure (54).

Controlled exposure studies support these epidemiological reports. The recent Multicenter Ozone Study in Older Subjects (MOSES) examined the respiratory responses to low concentrations of ozone in 87 healthy adults aged 60 years (55). Exposure to filtered air rapidly increased mean FEV_1_ and FVC values but ozone attenuated these increases in a dose-dependent manner. This effect persisted for 22 h after exposure. Ozone enhanced plasma CC16 levels and sputum neutrophilia in a dose-dependent manner after 4 h and sputum neutrophil counts correlated with airway biopsy counts (56). Sputum neutrophilia returned to normal levels 18 h post-ozone exposure (55, 56). Worryingly, concentrations of ozone close to the National Ambient Air Quality Standard (0.06 ppm) decreased lung function and increased airway inflammation in healthy young adults (57). This response was independent of a reduction in the expression of the anti-oxidant gene glutathione S-transferase mu 1 (GSTM1).

Acute ozone exposure (2 h, 0.4 ppm) of 9 healthy adults enhanced sputum neutrophils, monocytes and macrophages after 6 h with a significant upregulation of innate immune (mCD14, CD11b, CD16) and antigen presenting (CD86, HLA-DR) markers (58). Ozone also enhanced sputum interleukin (IL)-6 and induced a transient decline in lung function but had no effect on exhaled NO. This suggests that ozone can prime innate immune cells that may be important for susceptible subjects subsequently exposed to inhaled allergens.

It is unclear whether ozone acts independently of other environmental factors. In some studies ozone alone enhances plasma and sputum inflammatory cells and mediators (CC16, IL-6, and matrix metalloproteinase (MMP)9) (59) with no effect seen with carbon black ultrafine particles (cbUFP). However, other studies show no effect of ozone alone (60). Overall, the effect of ozone on immune cell numbers and activation are dependent upon the dose and timing of exposure and the samples collection point. This makes comparison of the studies more complex.

Sputum induction on 27 healthy adults before and after ozone exposure (0.4 ppm, 2 h) gave distinct transcriptional responses according to whether there was a significant increase in sputum neutrophils (61). These ozone responders had activated innate immunity (increased expression of CD16, CD11b, and CD80 and elevated IL-8 and IL-1β expression) with reduced immune cell trafficking pathways (Figure 1). These distinct signatures may explain the effects of ozone in sensitive individuals. Healthy subjects who respond to ozone also demonstrate significantly enhanced sputum levels of the miRNAs miR-132, miR-143, miR-145, miR-199a^*^, miR-199b-5p, miR-222, miR-223, miR-25, miR-424, and miR-582-5p (62). The predicted targets of these miRNAs included inflammatory and immune-response pathways.

Gene arrays of BAL cell mRNA isolated after exposure of combined healthy and asthmatic subjects to various doses of ozone were subjected to pathways analysis (63). This highlighted enhanced inflammation and repair pathways particularly those involved in chemokine/cytokine secretion, activity, and receptor binding; metalloproteinase and endopeptidase activity; adhesion, locomotion, and migration; and cell growth and tumorigenesis regulation. The response in asthmatics was 1.7–3.8-fold greater than that seen in healthy subjects. The highest differentially expressed up-regulated gene was osteopontin and treatment of an airway epithelial cell line with polymeric osteopontin enhanced wound closure in an α9β1 integrin-dependent manner.

Bioinformatic analysis of gene expression profiles has indicated that the impact of ozone exposure on generating acute lung injury (ALI) in animal models is significantly correlated with signatures seen in 3 human lung injury bronchoalveolar lavage datasets (*r* = 0.33–0.45, *p* <10^−16^) (64). Signatures relate to neutrophils, cytokine and chemokine activation and kinase activation pathways and there is a significant overlap of 181 potential drug targets common between human and animal models.

Metabolomics analysis of bronchoalveolar lavage fluid (BALF) from healthy subjects following a 2 h exposure to ozone (0.3 ppm) whilst undertaking light exercise could detect 28 differentially expressed metabolites after 1 h indicative of increased glycolysis and a feedback antioxidant response (65). In contrast, at 24 h post ozone exposure 41 differentially expressed metabolites were observed. These were associated with enhanced proteolysis and lipid membrane turnover indicating repair of airway tissues (65). Interestingly, whilst many human ozone exposure models include light exercise, the effect of ozone on exhaled oxidative stress markers were attenuated by physical activity in healthy adolescents (66).

## Sex Specific Responses to Ozone

Sex-specific differences exist in the incidence and prognosis of respiratory diseases with women having a greater risk of adverse health outcomes from air pollution than men (Figure 1). However, the underlying mechanisms for this are unknown although sex-specific patterns of immune gene expression and regulatory networks have been proposed (67). The expression of 84 inflammatory genes in murine lungs 4 h after ozone exposure (2 ppm, 3 h) was examined and linked to lung histology and bronchoalveolar lavage (BAL) cell counts (68). Inflammatory gene mRNA levels in female mouse lungs were significantly lower than in males for 20/72 genes analyzed following ozone exposure (3 h at 2 ppm) whilst only 4/72 were higher. Down-regulated genes included TLRs (TLR1, TLR3, TLR6), cytokines (IL1α, IL23α), chemokines (CXCL5, CCCL12, CCL11, CCL25), cytokine and chemokine receptors (CCR3, CCR2, CXCR4, IL1RAP), as well as other immune mediators, enzymes, receptors and transcription factors (C3AR1, CD14, CD40, RIPK2, PTGS2, NF-κB, LY96). Up-regulated pathways included macrophage activation (CXCL2, CCL19), Toll-like-receptor activation (MYD88), and modulation of IL-6 responses (C4B). Bioinformatic analysis indicated that immune cell adhesion and movement and pattern recognition receptor functions were enriched in females whereas the inflammatory response, cell-to-cell signaling and interaction, and cellular movement pathways were enriched in males. Overall, the results indicate a decrease in innate immune responses in females compared to males in response to ozone exposure (68).

The same group also examined miRNA lung expression and found that the baseline expression of miR-222-3p and miR-466 k were up- and down-regulated, respectively, as different in the lungs of male and female C57BL/6 mice which pathway analysis indicated controlled the expression of cell-to-cell signaling and interaction, cellular growth, proliferation, and gene expression (67). Exposure of male and female C57BL/6 mice to 2 ppm of ozone or filtered air for 3 h resulted in sex differences in the expression of 9 miRNAs including miR-130b-3p, miR-17-5p, miR-294a-3p, and miR-338-5p which are involved in inflammation and targeted key regulators of the immune response including IL-6, SMAD2/3, and TMEM9. The top networks included pathways associated with cell cycle, cellular development, growth, proliferation, and movement along with cell death and survival.

The detrimental effects of ozone in the lung have been attributed to bronchial-alveolar epithelial damage and defects in the bronchial-blood barrier but whether these effects are different between males and females is unclear. 3 h exposure to 2 ppm ozone up-regulated lung IL-6 levels in both males and females but the expression of IL-6R in the lung was only elevated in females (69). This was associated with a significant increase in STAT3-Y705 phosphorylation in both females and males whilst JAK expression and phosphorylation differed between sexes. In addition, NF-κB (p105/p50) and AKT1 protein levels were significantly increased only in ozone-exposed females. Furthermore, these responses varied across the oestrous cycle (69).

Ozone exposure following an infection results in greater survival in males compared to females whereas survival after infection alone is greater in females (70). Using a multianalyte immunoassay to measure 59 analytes in the lung at 4 h indicated that females had a greater oxidative stress response to ozone than males indicated by enhanced lung expression of EGF, fibrinogen, haptoglobin, IL-17, Interferon γ inducible protein (IP)-10, KC/CXCL1, MCP-5, M-CSF, MIP-2, RANTES, SCF, and TNF-α. This enhanced response was inversely correlated with the poorer survival (70). It is surprising how few studies take gender into account when modeling the effects of environmental pollutants.

Previous work from the Shore group has demonstrated that short-chain fatty acids (SCFAs) which represent the end products of bacterial fermentation are important in driving the airway response to ozone following changes in the mouse gut microbiome (71). The same group have reported that these ozone-induced responses are sex-dependent. Indeed, acute ozone exposure to ozone resulted in greater AHR in male compared to female mice which disappeared with antibiotic treatment (71). Female pups kept in the same cages as male pups also exhibited a greater AHR in response to ozone exposure which was mimicked by addition of the SCFA propionate to the drinking water. The data indicates that microbiome-derived SCFAs are processed in a sex-dependent manner to modify ozone-induced AHR.

## Ozone may Affect Inhaled and Oral Drug Responses

Acute inhalation of ozone in rodents modulates the expression of circulating stress hormones including adrenalin and corticosterone/cortisol and adrenalectomy attenuates ozone-induced lung injury and inflammation. This may account for the variable sensitivity seen to ozone *in vivo*. This also suggests that there is a potential feedback loop that may be impacted by exogenous drugs (β2-agonists and ICS) used to treat patients with asthma and COPD which affects the response to environmental ozone (72, 73).

High levels of IL-17A have been associated with a relative corticosteroid (CS)-insensitivity in mice (74). Chronic ozone exposure (2.5 ppm; 3 h twice weekly for 6 weeks) enhanced numerous inflammatory mediators including IL-17A, which was associated with neutrophil infiltration into the lung (75). Prophylactic treatment with a combination of dexamethasone (2 mg/kg) and an anti-murine IL-17A monoclonal antibody (IL-17mAb) reduced chronic ozone-induced neutrophilia and total inflammatory cells, inhibited BAL protein levels of IL-8, IL-17A, and TNF-α and the mean linear intercept (Lm) as a marker of emphysema. Combined administration also significantly elevated BAL interferon (IFN)γ levels and glucocorticoid receptor (GR) expression whilst supressing NF-κB and p38 MAPK phosphorylation (75). The study highlighted the critical role of IL-17A in reducing CS responses as a result of ozone exposure.

Many studies implicate oxidative stress in the pathophysiology of COPD and the relative corticosteroid-insensitivity observed in most of these patients (76, 77). Repeated ozone exposure (3 ppm, 3 h) twice a week for 6 weeks induces many features of COPD including emphysema and lung/airway inflammation (78). This was associated with enhanced expression of caspase-3 and apoptosis protease activating factor-1 in macrophages and in the airway and alveolar epithelium. IL-13, KC/CXCL1, caspase-3, and IFNγ mRNAs were increased whereas heme oxygenase-1 (HO-1) mRNA decreased. These effects of chronic ozone exposure were relatively insensitive to high dose (2 mg/kg) dexamethasone given prophylactically (79). These effects were generally unaffected by the CS-upregulated protein DUSP1/MKP-1 although a small impact effect on remodeling was observed.

Interestingly, there is also a link between ozone exposure and endogenous CS responses in animal models as evidenced by the effect of the 11β-hydroxylase inhibitor metyrapone (80). Ozone (0.8 ppm, 4 h) exposure evoked a metyropone-sensitive 2-fold increase in plasma corticosterone without affecting epinephrine levels. The effects of ozone on ghrelin or plasminogen activator inhibitor-1 were unaffected by metyrapone. The effect of chronic ozone on endogenous CS responses should be investigated.

## Murine Models of Ozone Exposure Reflect Human Epidemiological and Challenge Models

The effects of acute and chronic ozone exposure in man have been confirmed in animal models and details of the transcriptomic effects elucidated in these rodent models and in human cellular models (81). Acute ozone exposure (1 ppm, 1 h) in the mouse caused respiratory epithelial disruption with protein leak, BAL neutrophil recruitment, lung inflammation and AHR. Chronic exposure (1.5 ppm, 2 h, twice weekly for 6 weeks) amplified these acute effects of ozone and furthermore resulted in collagen deposition, greater epithelial injury with reduced epithelial barrier height, distended bronchioles, and enlarged alveolar space indicative of peribronchiolar fibrosis and emphysema (Figure 1).

Repeated exposure of C57BL/6 mice to ozone (0.8 ppm, 4 h/day) for 9 days resulted in the presence of an asthma-like phenotype with airway eosinophilia, mucus cell metaplasia and activation of type 2 innate lymphoid (ILC2) cells (82). Interestingly, ozone (3 ppm, 2 h) induced airway eosinophilia and IL-5 expression as well as neutrophilia in BALB/c but not C57BL/6 mice (83). ILC2s isolated from BALB/c mice exposed to ozone expressed greater mRNA levels for IL5 and IL13 than seen in C57BL/6 mice. Anti-Thy1.2 treatment abolished ozone effects on AHR that were restored by addition of ILC2s. Thus, the role of ILC2 cells in ozone-induced AHR and airways inflammation are strain dependent.

The effects of ozone on lung inflammation and AHR are biphasic and are mediated, at least in part, by distinct eosinophil populations (84). The acute 1-day effect of ozone is associated with the presence of mature airway-resident eosinophils in close proximity to parasympathetic nerves causing AHR. Four days after exposure, newly divided eosinophils were recruited to airways in a TNFα-dependent manner to suppress ozone-induced AHR. In this case, only airway eosinophils correlated with AHR indicating the importance of inflammation in distinct airway compartments.

As in man, the effects of ozone are age-dependent with adolescent and young adult animals being more susceptible to changes in ventilation and pulmonary injury/inflammation (85). Older rats had an inability to induce adaptation of ventilatory function in response to repeated ozone exposure and augmentation of lung function was most prevalent in young adult animals exposed to subchronic ozone. For example, BAL γ-glutamyl transferase activity and lung inflammation were only significantly enhanced after acute ozone in adolescent and young adult rats. The susceptibility of children to the detrimental effects of ozone exposure may be due to the need for airway and lung growth to continue after birth (86). Neurokinin signaling is involved in ozone-induced cell death and in male infant rhesus monkeys ozone increased neurokinin pathway expression in the conducting airways. In addition, ozone exposure at an early age resulted in enhanced inflammation and AHR.

Asthma exacerbations are often triggered by air pollution including ozone and in a murine model of asthma, ozone exposure resulted in enhanced AHR and greater neutrophilic inflammation associated with increased BAL levels of TNF-α, IL-13, and HA (87). Ozone also decreased epithelial cell density and increased mucus production. Furthermore, In BALB/c mice sensitized to ovalbumin, combined ozone (4 h, 0.5 ppm) and diesel exhaust particles (DEP, 4 h, 2 mg/m^3^) exposure once a week for 4 weeks exacerbated AHR (88). However, in this instance, enhanced AHR was not linked to pulmonary inflammation. This mimics the situation in man where there is often a disconnect between inflammation and AHR (54) (see above).

## Effects of Ozone on Innate Immune Cells in the Airway

Spleen-derived macrophages and inflammatory mediators are important in ozone toxicity (89). Splenectomy resulted in decreases in pro-inflammatory macrophages (CD11b+Ly6CHi) in the lung and down regulation of CCR2, CCL2, and CCL4, but increases in anti-inflammatory (CD11b+Ly6CLo) macrophages compared to ozone-exposed wild type mice. The numbers of CD11b+Ly6G+Ly6C+ granulocytic (G)-myeloid derived suppressor cells (MDSC)s were also reduced in ozone-exposed splenectomised mice. Importantly, changes in lung macrophage subpopulations and in lung G-MDSCs correlated with reduced ozone toxicity, BAL protein content and 4-hydroxynonenal expression. Ambient ozone exposure resulted in impaired antibacterial host defense, in part related to disruption of epithelial barrier and effective phagocytosis of pathogens (Figure 1). The functional response to ambient ozone is dependent on many components of the innate immune signaling (90).

Inverted formin-2 (INF2) is a significant quantitative trait locus that contributes to ozone-induced neutrophilic inflammation in mice (91). Transcriptomic and bioinformatic analysis of ozone-exposed INF2(-/-) and wild type mice revealed key roles for major histocompatibility complex (MHC) class II genes and the TNF gene cluster in ozone-induced lung neutrophilia.

The influx of distinct macrophage and monocyte subpopulations into the lung during acute ozone (0.8 ppm, 3 h)-induced lung injury was associated with CCR2 expression (92). There was a more predominant effect on proinflammatory CCR2+ and CD11b+LY6CHi and NOS2+ macrophages. However, there was also an effect on the timing of the recruitment of anti-inflammatory mannose receptor+ macrophages and CD11b+LY6CLo macrophages.

Ozone-induced γδ T cells are critical for the induction of alternatively activated M2 macrophages and the resolution of inflammation after ozone exposure (93). It is probable that secretion of IL-17A by γδ T-cells alters macrophage polarization and the clearance of apoptotic cells.

Furthermore, ozone exposure regulates the expression of galectin-3 (GAL3), which is a lectin that controls macrophage activity (94). Ozone increased the numbers of proinflammatory (GAL3+, NOS2+) and anti-inflammatory (MR1+) macrophages within the lungs. NOS2+ macrophage numbers were reduced in GAL3(-/-) mice whilst MR1+ macrophages were increased. GAL3(-/-) mice had reduced numbers of ozone-induced LY6Chi macrophages but no effect on LY6Clo macrophages was seen. In addition, the numbers of granulocytic (G) and monocytic (M) myeloid-derived suppressor cells (MDSC) were altered in GAL3(-/-) mice with G-MDSC being reduced and M-MDSCs being increased. These changes in immune cell populations correlated with tissue damage after ozone exposure.

Ozone-induced lung expression of GR-1+ macrophages expressing high levels of MARCO and CX3CR1 are not derived from circulating CCR2+ cells but from lung resident macrophages (95). This cell population was reduced in CXCR1(-/-) mice which had an enhanced AHR and inflammatory response to ozone. This suggests that this subset of lung macrophages protect the host from ozone (96).

## Effect of Ozone on Gene Expression in Animal and Cellular Models

Acute (1 week) and chronic (3 and 6 weeks) exposure to ozone (3 ppm, 3 h, twice weekly) elevated total BAL cell counts associated with increased chemokine and cytokine expression. This increased inflammation was associated with Lm (mean linear intercept) scores after chronic exposure (97). The expression and activity of nuclear factor (erythroid-derived 2)-like 2 (Nrf2), a marker of anti-oxidant activity, was present after 1 week but was lost at 3 and 6 weeks, at which time the expression and activation of HIF-1α was seen. HIF-1α-induced genes such as histone deacetylase (HDAC)2, vascular endothelial growth factor (VEGF), kelch-like ECH-associated protein (KEAP)1, and macrophage migration inhibitory factor (MIF) were also up-regulated at 3 and 6 weeks ozone exposure. This suggests that the loss of the antioxidative stress response and activation of the HIF-1α pathway contribute to the inflammatory response and emphysema observed in ozone-exposed mice.

**Figure 2:**
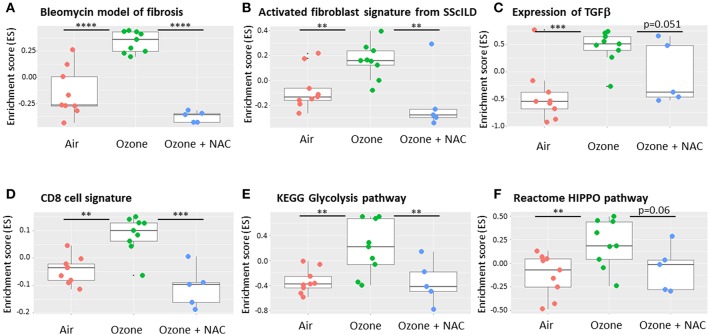
Effect of ozone and N-acetyl cysteine (NAC) on the enrichment of gene signature for fibrotic pathways within murine lungs. Using a bioinformatic technique called gene set variation analysis (GSVA) it is possible to interrogate transcriptomic arrays or RNA-sequencing data for the presence of gene signatures that are unique for specific pathways and or cell types. These signatures may be available from the literature or from online resources or be self-generated. The results show the enrichment scores (ES), a summary of the mRNA expression of all the genes in each signature across the whole data with a range of −1 to +1. Six weeks ozone exposure (2.5 ppm, 3 h/2 days a week) enriches for markers of fibrosis including bleomycin exposure **(A)**, activated fibroblasts **(B)** and transforming growth factor (TGF)β gene expression **(C)** compared with air. The analysis also reveals enrichment for individual cell types such as CD8+ T-cells **(D)** and key remodeling pathways such as the glycolysis **(E)** and HIPPO **(F)** pathways. The ES for each of these signatures is reversed by co-treatment with N-acetyl cysteine (NAC, 100 mg/kg i.p.). Data is obtained from the experiments reported in Yang et al. (83). Data are presented as individual data points with box-and-whisker plots showing median and interquartile range. ^**^p < 0.01; ^***^p < 0.001; ^****^p < 10^−5^.

Further analysis of the transcriptomic data from this model using gene set variation analysis (GSVA) (98, 99) and the online freeware R Bioconductor (https://www.bioconductor.org/) (Figure 2). GSVA is a non-parametric, unsupervised method for estimating the variation of sets of genes or pathways across a dataset and indicates that there is enrichment of gene signatures associated with fibrosis in this model that are reversed by co-treatment with N-acetyl cysteine (NAC). This approach also enables the analysis of other activated cells and pathways such as CD8+ T-cells, glycolysis and the HIPPO pathway that impact upon the mechanisms of chronic (6 week) ozone exposure on lung pathophysiology. However, not all models of repeated ozone challenge enhance inflammation, AHR and mucus hypersecretion in murine models of asthma (100). In this model, repeated ozone exposures reduced ovalbumin-induced AHR but did enhance mucus hypersecretion potentially via effects on immune dysregulation.

The effect of acute ozone exposure (0.25, 0.5, or 1.0 ppm for 4 h) on gene expression profiles were examined in male Wistar Kyoto rats. Acute inflammatory response genes along with genes involved in cell adhesion and migration, steroid metabolism, cell cycle control, and cell apoptosis/growth were up-regulated in an NF-κB/RELA, SP1, and TP53-dependent manner (101). Ozone treatment of rats (2 ppm, 3 h) enhances the expression of 8-hydroxy-2'-deoxyguanosine (8-OHdG) and of HO-1 in alveolar macrophages (AM) in a time-dependent manner (102). Ozone also induced markers of apoptosis (cleaved caspase-9) and of autophagy (beclin-1) in AM and enhanced BAL MMP-2 and MMP-9 expression. Enhanced NF-κB-associated inflammatory-type AM (MCP-1, NOS2, and COX-2) was also seen along with markers of anti-inflammatory/wound repair macrophages (arginase-1, YM1, and GAL3). Overall, this indicates that both proinflammatory/cytotoxic and anti-inflammatory/wound repair macrophages are activated in response to ozone in the rat. The authors suggested that these effects were downstream of ozone interactions with the airway lining fluid to oxidatively modify local lipids and proteins.

Exposure of BALB/c mice to ozone (2.5 ppm) for 6 weeks induced a chronic inflammatory process similar to that seen in COPD (78). These features included alveolar enlargement and damage linked to epithelial cell caspase 3-induced apoptosis, enhanced inflammation (IL-13, KC/CXCL1, and IFNγ), increased protease (MMP-12) expression and airway wall remodeling (enhanced collagen deposition). In C57BL/6J mice exposed to ozone before subsequent challenge with *Escherichia coli* LPS resulted in greater levels of total protein and of proinflammatory cytokines in lung lavage fluid and amplified the LPS-response in lung tissue (90). This was associated with enhanced systemic IL-6 expression. The enhanced LPS response following ozone exposure was due to enhanced macrophage TLR4 expression.

Ozone also affects epithelial cell tight junctions *in vivo* by suppressing the expression of claudins (CLDNs) (103). Chronic ozone exposure increased CLDN3, CLDN4, ROS, Nrf2, and Keap1 protein expression but decreased lung CLDN14 protein expression. This suggests that ozone may affect tight junction formation, at least in part, through a ROS-dependent mechanism. Ozone inhalation also suppresses mitochondrial gene expression, enhances mtDNA damage and affects mtDNA copy number indicating that the loss of pulmonary function and inflammation are linked with the loss of mtDNA integrity and DNA repair capability (104) (Figure 1).

The diabetes-prone KK strain of mice subjected to ozone (0.5 ppm, 5 h/day for 13 weekdays) had a further impaired insulin response linked to reduced plasma insulin and leptin levels (105). Ozone exposure for 3 weeks resulted in enhanced lung and systemic inflammation with increased numbers of monocytes/macrophages in both blood and visceral adipose tissue. Activation of CD4+T cells was also detected along with up-regulation of inflammatory (CXCL-11, IFNγ, TNFα, IL-12, and NOS2) and oxidative stress (COX4, COX5a, SCD1, NRF1, and NRF2) genes in visceral adipose tissue. The data indicates that repeated ozone exposure exacerbates insulin resistance, oxidative stress, and activates the innate immune system in susceptible animals.

Gene arrays using RNA from ozone (0.75 ppm, 2 h)-exposed primary HBECs indicated up-regulation of pro-inflammatory (e.g., IL-6, IL-8) and vascular function (e.g., PTGS2) pathways (106). In contrast, nitrogen dioxide-exposed cells gave a large oxidative stress response signal highlighting their differing mechanistic responses. In order to understand the mechanisms underlying the epidemiological evidence for an association between black carbon (BC) and ozone exposure with adverse health effects. The effects of BC and ozone-oxidized BC (OBC) on transcriptomic profiles in human lung epithelial A549 cells were studied (107). Only a few oxidative stress-related genes were co-regulated by BC and OBC whereas ~40% of inflammatory genes and 33% of autophagy-related genes were identical. This highlights differences in the toxic mechanisms between different environmental pollutants but does indicate a degree of co-operation in modulating inflammation above oxidative stress.

A combination of structural cells including epithelial cells and resident immune cells such as macrophages are critical for the lung innate immune response to ozone. Co-culture of human airway epithelial cell lines and BAL macrophages from healthy subjects have been exposed to ozone (108). Macrophages had elevated levels of alternative activation markers, enhanced cytotoxicity and reduced phagocytosis compared to those treated in monoculture. Co-culture also affected the ability of macrophages to regulate HA expression. These results highlight the importance of cell-cell communication and particularly epithelial-derived factors in modulating ozone-induced macrophage immunophenotypes.

Exposure of primary airway epithelial cells from juvenile rhesus monkeys to ozone resulted in attenuated IL-6 and IL-8 expression but this effect was altered if the animals had also been challenged with LPS (109). In accordance with these results, the expression of miRNAs such as miR-149 that control IL-6 expression were also dysregulated. The authors suggested that early-life exposure to ozone reprogrammed the innate immune response of airway epithelial cells to subsequent bacterial challenges in later life.

## Cytokine Mediators of Ozone Effects

IL-33 is critical in mediating ozone effects in murine models (Figure 1). For example, co-exposure of mice to BC and to OBC enhances inflammation and lung damage compared to individual exposures (107). Neutralizing antibodies against IL-33 prevented BC/OBC-induced lung damage and inflammation through MAPK- and PI3K-AKT-dependent pathways (110). Interestingly, anti-IL-6 antibodies had no effect on these ozone-induced parameters although anti-IL-33 did suppress IL-6 expression.

Furthermore, ST2- and IL-33-deficient mice exposed to ozone (1 ppm, 1 h) gave a further reduction in epithelial cell injury, myeloid cell recruitment, and inflammation (111). In contrast, the tight junction proteins E-cadherin and zonula occludens 1 (ZO-1) and neutrophil ROS and AHR are diminished. ST2 neutralization mimicked these effects and highlighted the importance of IL-33/ST2 signaling in epithelial barrier function and inflammation following ozone exposure. Interestingly, the enhanced effects of ozone exposure on inflammation and AHR seen in obese mice was also dependent on IL-33 (112). This was due to the combined effect of obesity and ozone to promote T2 cytokine production from ST2+ γδ T cells and ILC2 cells.

Administration of an anti-IL17 mAb to chronic ozone-exposed mice results in suppression of BAL KC/CXCL1, BAL total cell and neutrophil counts and of AHR (113). Anti-IL-17 also reduced p38 MAPK activation and highlighted the important role of IL-17 in ozone-induced inflammation, lung injury and AHR. This result using an anti-IL-17 mAb augments previous data using IL-17A and IL-1R1 knockout mice which demonstrated that ozone-induced IL-17A and neutrophilic airway inflammation was downstream of the caspase-1-IL-1 pathway (114). Prolonged (7 h) low-dose (0.7 ppm) ozone exposure resulted in neutrophilic airway inflammation, accompanied by an increased production of IL-1β, IL-18, IL-17A, Granulocyte-colony stimulating factor (G-CSF), INFγ-inducible protein 10 (IP-10) in BAL which were attenuated in IL-17(-/-) mice. Furthermore, IL1R1(-/-) mice as well as mice given a caspase inhibitor *in vivo* show decreased IL-17A expression and airway inflammation. Although IL-17A is important for ozone-induced AHR it does not appear to be critical for the lung destruction seen in IL-17A(-/-) mice (115). These mice also failed to show an effect of IL-17A on airway inflammation.

The IL-1/inflammasone axis has been associated with ozone-induced airway inflammation in allergic asthmatic patients (116) and both IL-1α(-/-) knockout mice and blocking antibodies provide evidence for a role for IL-1 in ozone-induced epithelial cell barrier function, inflammation, and AHR (117). These prevented ozone-induced effects via IL-1R1 and the adaptor protein MYD88. IL-1 release is downstream of inflammasome activation and evidence suggests that ozone-enhanced lung oxidative stress causes inflammasome activation and IL-1 release eventually leading to alveolar destruction/emphysema and respiratory failure (118). In contrast, ozone appears to suppress NLRP3-mediated inflammation by enhancing NRF2 activity in rats (119).

## Conclusion

Ozone induces the production of reactive oxygen species (ROS) within epithelial lining fluid, which in turn activates ROS-related intracellular signaling pathways within airway epithelial cells resulting in enhanced expression of inflammatory and remodeling factors. Analysis of transcriptomic profiles in human and mouse tissues and cells have indicated the key role of specific immune and inflammatory pathways in driving the detrimental effects of ozone exposure. These data have indicated treatable mechanisms (p38 MAPK, TLRs, NRLP3, HIPPO) and mediators (IL-1, IL-17, and IL-33) that may be suitable for ameliorating the effects of ozone. Biologics may provide a suitable means of prolonged treatment in susceptible individuals where ozone exposure is high and enduring.

Future analysis of ozone effects needs to incorporate more global assessments of inflammation, AHR, and epithelial damage. The advent of increasingly affordable bulk and single-cell RNA-sequencing will provide insight into ozone actions particularly when applied to lung/airway samples from both healthy subjects and patients with disease. Recent epidemiological analysis of ozone effects in adults and children should be linked to real-time monitoring of individual exposures linked to cell/tissue sampling. Appreciation of the mechanisms by which ozone exerts its effects suggest that potent drugs that target TLRs or redox-sensitive kinases to restore immune skewing induced by ozone as well as restoration of metabolic perturbations may be of use when exposures cannot be reduced to “safe” levels. Indeed, it is likely that even current levels that are considered safe may have adverse effects on both healthy subjects and patients with different airways diseases. This will require public health changes and government interventions on a global level. This is likely to become particularly important with temperature changes associated with climate change. Ozone has profound effects on the airway, particularly in sensitive subjects, and although drugs may be developed that prove effective it is better to prevent the exposure at all ages.

## Author Contributions

IA and KC conceived the topic. IA and SM drafted the manuscript. IA, KC, and SM edited and revised the manuscript.

### Conflict of Interest Statement

The authors declare that the research was conducted in the absence of any commercial or financial relationships that could be construed as a potential conflict of interest.
